# Dietary Fatty Acids and Changes in Blood Lipids during Adolescence: The Role of Substituting Nutrient Intakes

**DOI:** 10.3390/nu9020127

**Published:** 2017-02-11

**Authors:** Carla Harris, Anette Buyken, Sibylle Koletzko, Andrea von Berg, Dietrich Berdel, Tamara Schikowski, Berthold Koletzko, Joachim Heinrich, Marie Standl

**Affiliations:** 1 Institute of Epidemiology I, Helmholtz Zentrum München—German Research Centre for Environmental Health, 85764 Neuherberg, Germany; carla.harris@helmholtz-muenchen.de (C.H.); heinrich@helmholtz-muenchen.de (J.H.); 2 DONALD Study, IEL-Nutritional Epidemiology, University of Bonn, 44225 Dortmund, Germany; buyken@uni-bonn.de; 3 Ludwig-Maximilians-University of Munich, Dr. von Hauner Children’s Hospital, 80337 Munich, Germany; sibylle.koletzko@med.uni-muenchen.de (S.K.); Berthold.Koletzko@med.uni-muenchen.de (B.K.); 4 Department of Pediatrics, Marien-Hospital Wesel, 46483 Wesel, Germany; avb.rodehorst@gmx.de (A.v.B.); berdel.vonberg@t-online.de (D.B.); 5 IUF-Leibniz Research Institute for Environmental Medicine (IUF), 40225 Düsseldorf, Germany; tamara.schikowski@iuf-duesseldorf.de; 6 Institute and Outpatient Clinic for Occupational, Social and Environmental Medicine, Inner City Clinic, University Hospital of Munich (LMU), 80336 Munich, Germany

**Keywords:** fatty acids, lipids, isocaloric substitution, diet, carbohydrates, adolescence, epidemiology

## Abstract

The relevance of dietary fatty acids (FA) for blood lipids should be assessed in the context of substituting nutrients. Such evidence is lacking for adolescents. This study describes prospective associations of dietary FA with changes in serum lipids during adolescence, and considers the theoretical isocaloric replacements of saturated FA (SFA) with other FA or carbohydrates (CHO). Children from the GINIplus and LISAplus birth cohorts, with data on FA intakes (at age 10 years) and serum lipids (at age 10 and 15 years), were included (*n* = 1398). Associations of SFA, monounsaturated FA (MUFA), *n*-3 polyunsaturated FA (*n*-3 PUFA) and *n*-6 PUFA, with changes in low-density lipoprotein (LDL), high-density lipoprotein (HDL), triglycerides (TAG), and total cholesterol to HDL ratio (TOTAL:HDL), were assessed by linear regression. Substitution models assessed isocaloric replacements of SFA with MUFA, *n*-3 PUFA, *n*-6 PUFA or CHO. Higher SFA intakes were associated with decreasing TAG. No associations were observed for fatty acid intakes with LDL, HDL or TOTAL:HDL. In females, replacing SFA with CHO was associated with increasing LDL, TAG and TOTAL:HDL. Our findings confirm observations in adults, although sex-specific determinants seem relevant in our adolescent population. Overlooking the nutrient context when limiting SFA intakes might have detrimental consequences appreciable as early as adolescence.

## 1. Introduction

Since the first appearance of evidence suggesting a detrimental role of saturated fatty acids (SFA) in the development of coronary heart disease [1,2], the advice to reduce SFA consumption has become a major component of health-promoting strategies [3]. Nevertheless, inconsistent findings among emerging studies have led scientists to question the independent association of SFA with the development of cardiovascular disease (CVD) [4,5,6]. It has become clear that evidence supporting a reduction of SFA intake must be interpreted in the context of the specific nutrients consumed in its place [7,8]. In 2008, the FAO and the WHO stated convincing evidence for an improved lipoprotein profile in adults when replacing SFA with polyunsaturated fatty acids (PUFA) and, to a lesser extent, with monounsaturated fatty acids (MUFA). On the other hand, replacing SFA with carbohydrates (CHO) was reported to reduce low-density lipoprotein (LDL) but also high-density lipoprotein (HDL) levels [9]. 

It is currently well established that CVD risk factors progress from childhood and adolescence into adulthood [10]. Results from numerous longitudinal cohort studies have indicated strong tracking of serum lipids from childhood to adulthood [11,12,13]. Considering the implications this can have for later disease development, improving our understanding of the role of dietary fatty acid intakes in children is of major importance for the early implementation of dietary advice. The period concerning pubertal development is of interest due to the rapid growth and development as well as behavioral changes occurring at this stage [14,15]. However, despite the growing evidence in adults [16,17,18], the amount of reliable and comparable data on dietary fatty acid intakes in children and adolescents is scarce [19]. Studies observing the associations of total [20,21,22] and saturated fat [23,24] with blood lipid concentrations have reported mixed results. In particular, longitudinal studies on the theoretical implication of different replacements of SFA on lipid profiles in children and adolescents are lacking. A 2002 study using repeated measures at ages 8 and 11 years, suggested associations with serum lipids similar to those observed in adults when replacing SFA with MUFA or PUFA [25]. Further studies are required to learn whether such associations persist during the period of pubertal development.

The current study therefore aims to describe the prospective associations of fatty acid intakes during childhood with changes in serum lipid concentrations during adolescence. Furthermore, we are interested in observing how associations with SFA may depend on the choice of substituting nutrient. We therefore consider changes in blood lipids following the theoretical reduction of SFA in the context of different isocaloric replacements with other fatty acids or with carbohydrates.

## 2. Materials and Methods

### 2.1. Participants

The present study used data from the 10- and 15-year follow-up assessments of the ongoing GINIplus (German Infant Nutritional Intervention plus environmental and genetic influences on allergy development) and LISAplus (Influence of Lifestyle-Related Factors on the Immune System and the Development of Allergies in Childhood plus the Influence of Traffic Emissions and Genetics) birth cohort studies. Healthy full-term newborns were recruited from obstetric clinics in four German cities. Information was collected using identical questionnaires and at physical examinations. The study designs, recruitment and exclusion criteria have been described previously [26,27]. For both studies, approval by the local ethics committees (Bavarian General Medical Council, University of Leipzig, Medical Council of North-Rhine-Westphalia) and written consent from participants’ families were obtained.

### 2.2. Dietary Intake

Dietary intake data were collected at the 10-year follow-up assessment, using a self-administered food frequency questionnaire (FFQ) designed to assess food and nutrient intake over the past year in school-aged children, and validated to estimate energy, fatty acid and antioxidant intake [28]. In brief, subjects were asked to report estimated frequency and portion size of intakes of 80 food items. A quality control procedure was applied based on recommendations by Willett et al. for data cleaning in nutritional epidemiology [29,30]. Total daily energy intake and the intakes of SFA, MUFA, *n*-6 and *n*-3 PUFA, protein, carbohydrate and alcohol were calculated (in kcal/day) based on the German Food Code and Nutrient Database (BLS) version II.3.1 [31]. Each nutrient was expressed as its percentage contribution towards total daily energy intake (%EI), calculated as the ratio of energy from each nutrient to total daily energy intake, multiplied by 100. 

### 2.3. Blood Lipids

Blood samples were obtained during the 10- and 15-year follow-up physical examinations. The concentrations (mmol/L) of total cholesterol, LDL, HDL, and triglycerides (TAG) were measured in serum using homogenous enzymatic colorimetric methods on a Modular Analytics System from Roche Diagnostics GmbH Mannheim according to the manufactures instructions. External controls were used in accordance with the guidelines of the German Society of Clinical Chemistry and Laboratory Medicine. The ratio of total to HDL cholesterol (TOTAL:HDL) was calculated by dividing total cholesterol by HDL.

### 2.4. Statistical Analyses

**Figure 1:**
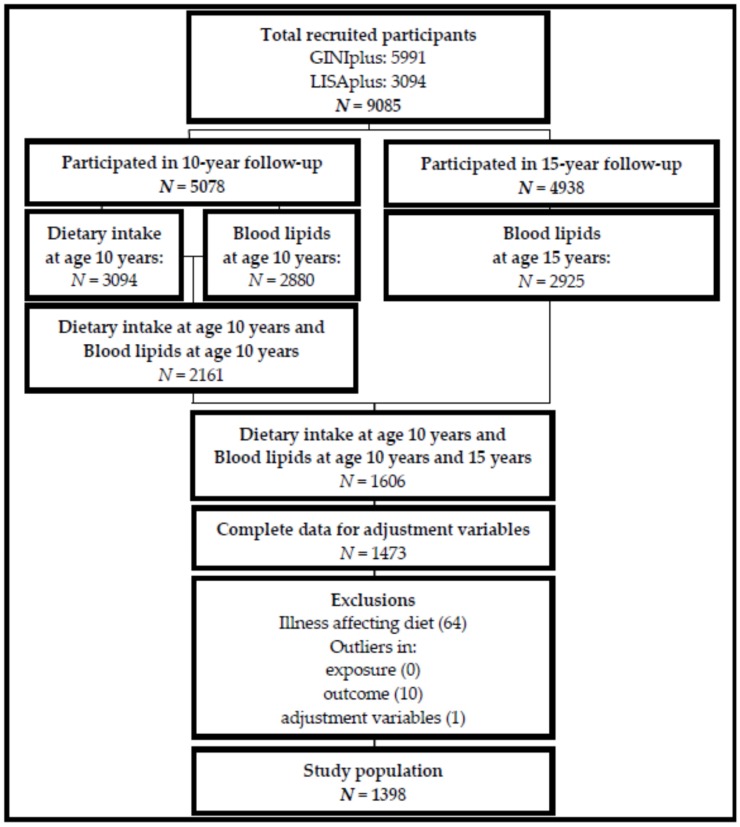
Study participants Dietary intake: intakes of fatty acids (saturated, monounsaturated, *n*-3 polyunsaturated, *n*-6 polyunsaturated), carbohydrate, protein, and alcohol obtained from FFQ; Blood lipids: low-density lipoprotein (LDL), high-density lipoprotein (HDL), triglycerides; adjustment variables: study, region, age, fasting status, BMI, screen-time, total energy intake, lipid concentration at age 10 years, parental education and pubertal onset; Illness affecting diet: e.g., diabetes, anorexia, coeliac disease, cancer, or medical dietary indications (e.g., gluten-free, lactose-free diets).

Participants with complete data on FA intakes at age 10 years, serum lipids at age 10 and 15 years, and all adjustment variables were included in the study (Figure 1). To test for differences due to attrition bias, we compared characteristics of participants lost to follow-up (data only available for exposure and outcome at 10 years) to those included in the present study analyses, who adhered at follow-up (data available for exposure at 10 years and outcome at 10 years and 15 years). Categorical variables, presented as percentages, were tested by Fisher’s exact test (binary variables) or Pearson’s Chi-squared test (variables with more than 2 levels). Continuous variables, presented as means (standard deviation), were tested by Student’s *t*-test. 

Statistical analyses were carried out in the total population and stratified by sex. Subject characteristics at ages 10 and 15 years were described by medians (25th percentile; 75th percentile) or counts (%). Differences from 10 to 15 years were tested using paired Wilcoxon signed rank test for continuous variables and McNemar’s χ^2^-test for categorical variables. Differences in characteristics between males and females at each assessment were tested using Wilcoxon signed rank test for continuous variables and χ^2^-test for categorical variables (Fisher’s exact test for binary variables). Changes (∆) in lipid concentrations and in TOTAL:HDL ratio were calculated by subtracting each measurement at the 10-year follow-up from its respective measurement at the 15-year follow-up. 

Using linear regression, two modelling approaches were applied. First, single nutrient models were fit to observe the changes in blood lipids when increasing habitual intakes of a single nutrient at a constant energy intake. Intakes of different fatty acids assessed at age 10 years were considered as the exposures of interest. Separate regression models were run for each exposure (SFA, MUFA, *n*-3 PUFA or *n*-6 PUFA) with the different blood lipid parameters (∆LDL, ∆HDL, ∆TAG, ∆TOTAL:HDL). Through this prospective approach, we aim to avoid any misleading findings emerging from the possible bidirectional relationship between fatty acid intakes and blood lipids assessed at a single time-point only. Second, substitution models were fit to observe the effect of replacing SFA with other fatty acids (MUFA, *n*-6 PUFA and *n*-3 PUFA) or with CHO, on the different blood lipid parameters (∆LDL, ∆HDL, ∆TAG, ∆TOTAL:HDL). These models included the exposure nutrient of interest as well as all other energy-bearing nutrients except SFA (the nutrient being “replaced”). In this way, energy intakes of protein, carbohydrate, alcohol and other fats are held constant; and by additionally including total energy intake in the model it is possible to interpret the resulting coefficients for each nutrient as its theoretical substitution for an equal amount of energy (%EI) from saturated fat, being the only energy-bearing nutrient not accounted for in the model. All models were adjusted for potential covariates in two steps. First, we adjusted for basic covariates (M_BASIC_): study (GINI observation arm; GINI intervention arm; LISA), recruitment region (Munich; Wesel; Bad Honnef; Leipzig), sex (male; female)—not in sex-stratified models—exact age at 10-year blood sampling (years), fasting status at blood sampling (not fasted (46%); fasted at one assessment (45%); fasted at both assessments (9%)), BMI (kg/m^2^) at age 10 years, screen-time (daily hours spent on activities in front of a screen: ≤2 h = low; >2 h = high) at age 10 years, total energy intake (kcal/day) at age 10 years, and lipid concentration (mmol/L) at age 10 years. In a second step, models were further adjusted for other potential confounders (M_ADJ_): parental education level (highest level achieved by mother or father: ≤10th grade = low/medium; >10th grade = high) and pubertal onset at age 10 years (oestrogen ≥ 18.5 pmol/L or testosterone ≥ 0.1 nmol/L = yes; oestrogen < 18.5 pmol/L or testosterone < 0.1 mmol/L = no). Given the high intercorrelation typically present amongst dietary components [32], we calculated correlation coefficients between pairs of nutrient variables, using Pearson’s product-moment correlation coefficient. A high negative correlation was observed between MUFA and CHO. By linearly regressing MUFA onto CHO and vice-versa, we computed residuals (MUFA_RESID_ and CHO_RESID_), which were uncorrelated with each other [33]. In order to avoid multicollinearity, these were included in the models as a stand-in for the original variable only when acting as a covariate (i.e., when assessing the effect of replacing SFA with CHO, CHO was included in its original form as the main predictor variable, and MUFA_RESID_ was included in place of MUFA, along with all other covariates, and vice versa). Results from the linear regression analyses are presented as regression coefficients (β) per interquartile range (IQR) increase in the relevant exposure variable, along with their 95% confidence interval (95% CI). A two-sided α-level of 5% was considered significant for the total population analyses. For the sex-stratified analysis we corrected for multiple testing using Bonferroni correction: the α-level was divided by 2 (2.5%) as the dataset was analyzed by sub-groups of two levels (male/female). Statistical analyses were conducted using R (www.r-project.org), version 3.3.0 [34].

## 3. Results

### 3.1. Study Population

The present analyses comprised of 1398 participants (681 females and 717 males). The derivation of the study population is presented in Figure 1. Subjects providing complete dietary intake data at age 10 years, measures of serum lipids at both 10 and 15 years, as well as information on all adjustment variables, were included (*n* = 1473). Differences in descriptive characteristics between participants included in the analyses and participants lost to follow-up are presented in the Table S1). Participants were excluded if they reported an illness affecting diet (e.g., diabetes, anorexia, coeliac disease, cancer) or medical dietary indications, such as gluten-free or lactose-free diets (*n* = 64). Clear outliers in blood lipid concentrations (*n* = 10), or adjustment variables (*n* = 1) were visually identified using descriptive plots and excluded from the analyses. Basic characteristics of the study population at age 10 and 15 years are described in Table 1. Both sexes had higher levels of LDL and HDL and lower levels of TAG and TOTAL:HDL at age 15 years compared to 10 years. Significant differences over time were observed for BMI, fasting status at blood sampling, screen-time and total daily energy intake, with higher values at age 15 years in both sexes (except energy intake, which decreased in females). Males reported higher screen-time and daily energy intake than females at both time-points, as well as higher fat and protein intakes at age 15. On the other hand, females at age 15 years reported higher carbohydrate intakes. Overall, most participants were from Munich (57.7%) with a high parental education (71.3%). Notably, more females than males had reached pubertal onset at the age of 10 years (74.4% females vs. 24.1% males).

### 3.2. Single Nutrient Models

The prospective associations of dietary fatty acid intakes (in %EI) at age 10 years with changes in serum lipid concentrations from age 10 to age 15 years are described in Table 2. Values are presented for basic (M_BASIC_) and fully adjusted models (M_ADJ_). The resulting β-coefficients indicate the changes in blood lipid concentrations (mmol/L) per IQR increase in the %EI of a given fatty acid, while maintaining total energy intake constant. A significant inverse association was observed between the intake of SFA (IQR increase in %EI) at age 10 years and the change in TAG concentrations from age 10 to age 15 years (M_ADJ_: β = −0.038 (95% CI = −0.075; −0.001), *p*-value = 0.042). A similar association was observed in females only, which was borderline statistically significant when corrected for multiple testing (M_ADJ_: −0.053 (−0.100; −0.007), *p*-value = 0.025). No associations were observed for any of the fatty acid exposures with the other assessed blood lipid parameters. 

### 3.3. Substitution Models

Table 3 shows the prospective associations of different dietary fatty acid intakes and CHO at age 10 years with changes in serum lipid concentrations from age 10 to age 15 years, when considering their theoretical substitution for SFA. Values are presented for basic (M_BASIC_) and fully adjusted models (M_ADJ_). Coefficients (β) obtained from these models represent an isocaloric substitution, i.e., the change in blood lipid concentrations when theoretically replacing the intake of SFA with another (specific) fatty acid or CHO, while maintaining total energy intake constant. A direct association was observed in the basic model for the substitution of CHO (IQR increase in %EI) for SFA with ∆LDL (M_BASIC_: 0.063 (0.000; 0.127), *p*-value = 0.05). Sex-stratified analyses indicated significant associations in females only, after correction for multiple testing: direct associations were observed for the substitution of CHO (IQR increase in %EI) for SFA with ∆LDL (M_ADJ_: 0.125 (0.021; 0.229), *p*-value = 0.019), ∆TAG (M_ADJ_: 0.098 (0.020; 0.176), *p*-value = 0.014), and ∆TOTAL:HDL (M_ADJ_: 0.115 (0.015; 0.215), *p*-value = 0.024).

## 4. Discussion

The present study used data from two large German birth cohorts to assess the prospective associations of fatty acid intakes with changes in blood lipid concentrations during adolescence. We found that higher intakes of SFA at age 10 years were associated with decreasing TAG between ages 10 and 15 years. Furthermore, we observed that the consumption of CHO at the expense of SFA in females was associated with increasing LDL, TAG and TOTAL:HDL. 

### 4.1. Single Nutrient Model

Our findings regarding the prospective association of SFA intakes with reduced TAG concentrations are in line with existing literature in adults [35]. The observed relationship might be considered somewhat counterintuitive, given the suggestions for a possible detrimental role of SFA in the development of coronary heart disease [1,2]. Conflicting evidence has been reported in younger populations, although existing studies are scarce and heterogeneous in terms of design, statistical methods and outcome measurements. A study in children aged 6 to 12 years, reported a positive association between SFA and total cholesterol concentrations [36], whereas an inverse association was observed in a study in 15-year-olds [37]. Other studies have reported no association between dietary fatty acids and blood lipids in pre-pubertal and pubertal children [24,38]. The often-observed association of SFA consumption with increased LDL in adults [35] was not present in our adolescent population. In fact, we observed a negative relationship in our total study sample, although the association did not reach statistical significance (*p*-value = 0.068). Although the evidence for an association of SFA with LDL has been widely accepted, recent studies in adults have emerged, reporting no association between SFA and CVD risk [4]. Nonetheless, the present results should be interpreted in the context of possible correlations among different nutrients. In our dietary data, SFA was highly positively correlated with MUFA (*r* = 0.77), and also presented strong negative correlations with CHO (*r* = −0.81), which limits the ability to disentangle the individual effects of SFA [32]. In light of this and the inverse association observed between SFA and TAG levels in the current study, we speculate that increasing the intake of SFA might have led to decreasing TAG levels indirectly through a reduction in CHO intake. Indeed, CHO, in particular simple sugars, have been shown to have a detrimental impact on blood lipids through raising TAG levels [39]. This has been suggested to result mainly from increased hepatic secretion of very-low-density lipoprotein (VLDL) as well as impaired plasma TAG clearance, possibly induced by reduced insulin sensitivity [40].

### 4.2. Substitution Model

Results from our substitution analyses showed that replacing SFA with CHO was associated with increasing LDL, TAG and TOTAL:HDL in females. Our findings are in line with studies in adults which report a detrimental effect on blood lipids when substituting CHO for SFA [41]. However, the specific effects on blood lipid parameters differed from those observed in adults, who typically present lower LDL levels [9,42,43] occurring in parallel with decreasing HDL levels, and having no effect on the TOTAL:HDL ratio [44]. An increase in LDL is, however, plausible if we consider results from randomized controlled trials which have reported positive linear associations of CHO with small-dense LDL [45]. Other studies have shown positive relationships between dietary sugars with plasma LDL and TAG [46]. In agreement with this and other studies [8,9,41,44] females in our study population also presented positive associations between CHO and TAG. This relationship can be attributed to the increased secretion of VLDL and impaired plasma TAG clearance described above. The greater number of VLDL particles in the blood could also have led to the increase in the LDL production rate [40], which was further reflected by the lower TOTAL:HDL ratio observed amongst our findings. A previous study, including a subset of our study population, showed that the highest contribution towards total energy intake at age 10 years came from “refined grains” and “sugar-sweetened foods” [30], which might suggest that the CHO consumed by children in our study population consisted largely of refined grains and sugars. Investigation into the effects of replacing SFA with different quality CHO is beyond the scope of the present study, but should be considered for further research in this age group. 

Comparison of our results with existing evidence in adolescents is restricted due to the absence of studies carried out during this life period. One similar study observed theoretical effects of substituting MUFA or PUFA for SFA, and total fat for CHO from ages 8 to 11 years [25]. The study findings included slightly lowered total cholesterol when replacing SFA with MUFA or PUFA and higher HDL when replacing CHO with fat. Based on these findings, the authors suggested a similar effect of diet on serum lipids to that observed in adults [25]. Unfortunately, LDL, TAG and TOTAL:HDL were not included among the serum lipid measurements and so comparison with our study is limited. Nevertheless, our findings also seem to be to some extent comparable to observations in adult populations. Our results further suggest a sex-specific role of SFA (when replaced by CHO), acting mainly in female adolescents. The reasons for this gender discrepancy are unknown but we speculate that it could be related to possible sex differences in dietary patterns, hormones or pubertal stage. A greater proportion of girls in our study had entered pubertal onset at age 10 years (Table 1). It has been shown that physiological insulin resistance occurs during puberty [47], which may explain why females in the present study were more vulnerable than boys to the potentially adverse effects of carbohydrates. Additionally, girls in our study had slightly higher CHO intakes, which persisted at a high level, whilst they decreased in boys.

### 4.3. Strengths and Limitations

One of the main strengths of this study is its focus on the prospective role of dietary fatty acids on blood lipids during adolescence, a life period not often addressed and becoming increasingly relevant in terms of later disease development. Furthermore, we consider isocaloric replacements of SFA, which can contribute toward better understanding its independent role in the context of other nutrients. For our analyses, we benefited from a large homogenous population of females and males, providing data covering a five-year period from childhood to adolescence. The longitudinal nature of this study is a key aspect which allows us to add to the limited knowledge regarding fatty acid intake and prospective changes in markers of cardiometabolic risk during adolescence. Given the observational nature of the study, causality cannot be implied; nevertheless, the prospective analysis offers a temporal component which provides stronger grounds for a causal interpretation. Whether the observed effect sizes in this study can be considered clinically relevant might be a point for discussion. Furthermore, considering that children in the present study are not a high risk population and present normal blood lipid levels, the observation of associations at this stage provide only an indication of a possible early role of dietary nutrients in the long-term development of CVD risk factors. Nevertheless, given the increasing evidence for the progression of risk factors from childhood to adulthood, preventive measures might already consider this age group. Our findings provide a relevant indication of possible dietary targets which could support the development of recommendations for early disease prevention.

A main drawback in nutritional epidemiology is the high intercorrelation amongst different nutrients, which, if overlooked, can lead to incorrect conclusions. The use of substitution models can provide additional insight through the adjustment for other nutrients. However, the method can result in multicollinearity within statistical models, again generating misleading associations [32]. In our analyses, we tackle this problem by residualizing highly correlated variables, allowing the new variable to be included in the same model as the previously correlated nutrient, while avoiding multicollinearity [33]. A further limitation in the present study was non-random loss-to-follow-up, which meant that, for example, children of lower social classes might be underrepresented in our analyses. Therefore the generalizability of our findings is limited, as these cannot be considered representative of the study area (Table S1). Finally, we are aware of problems associated with misreporting of dietary intake with the use of FFQs. However, the FFQ was validated to estimate fatty acids and antioxidants in school-aged children. We observed plausible values in terms of energy intake and believe that any misreporting was likely detected through extensive quality control, which was done at the expense of reducing the sample size, but with no substantial loss of power. 

## 5. Conclusions

In conclusion, our findings suggest that higher SFA intakes might lead to reductions in TAG concentrations during adolescence. We highlight that observed associations in this context are not independent of other correlated nutrients. Furthermore, replacement of SFA with CHO in female children is associated with increasing levels of LDL, TAG and TOTAL:HDL during adolescence. Our findings confirm observations in adult populations, where detrimental aspects of increased consumption of CHO at the expense of SFA have been reported. Sex-specific determinants may however play a greater role during adolescence. It is important that recommendations to reduce SFA intakes do not overlook the possible effects of other nutrients consumed in their place. 

## Figures and Tables

**Table 1:** Basic characteristics of the study population.

Variables	Total (*N* = 1398)			Females (*N* = 681)			Males (*N* = 717)		
	10 Years	15 Years	*p*-Value ^a^	10 Years	15 Years	*p*-Value ^a^	10 Years	15 Years	*p*-Value ^a^
**Blood lipids**									
LDL (mmol/L)	2.1 (1.7; 2.5)	2.3 (1.9; 2.7)	<0.01	2.1 (1.8; 2.5) ^†^	2.4 (2.0; 2.9) ^†^	<0.01	2.0 (1.7; 2.5)	2.2 (1.8; 2.6)	<0.01
HDL (mmol/L)	1.2 (1.1; 1.4)	1.5 (1.2; 1.7)	<0.01	1.2 (1.1; 1.4)	1.6 (1.4; 1.8) ^†^	<0.01	1.3 (1.1; 1.5) ^§^	1.4 (1.2; 1.6)	<0.01
TAG (mmol/L)	1.2 (0.9; 1.6)	1.0 (0.8; 1.4)	<0.01	1.2 (0.9; 1.6)	1.0 (0.8; 1.3)	<0.01	1.1 (0.8; 1.6)	1.0 (0.7; 1.4)	<0.01
TOTAL:HDL	3.8 (3.2; 4.5)	2.9 (2.5; 3.4)	<0.01	3.9 (3.4; 4.6) ^†^	2.9 (2.5; 3.4)	<0.01	3.6 (3.2; 4.4)	3.0 (2.5; 3.5) ^§^	<0.01
**Fatty acids**									
SFA (%EI)	12.6 (10.9; 14.7)	12.7 (10.8; 14.7)	0.621	12.5 (10.7; 14.7)	12.6 (10.6; 14.6)	0.190	12.8 (11.1; 14.8)	12.9 (10.9; 14.9)	0.512
MUFA (%EI)	10.7 (9.3; 12.3)	10.8 (9.4; 12.3)	0.133	10.7 (9.2; 12.1)	10.5 (9.1; 12.2)	0.608	10.7 (9.5; 12.4)	11.2 (9.6; 12.6) ^§^	<0.01
*n*-3 PUFA (%EI)	0.54 (0.49; 0.62)	0.56 (0.49; 0.65)	<0.01	0.55 (0.49; 0.63)	0.57 (0.49; 0.64)	0.009	0.54 (0.48; 0.62)	0.56 (0.48; 0.65)	<0.01
*n*-6 PUFA (%EI)	3.7 (3.2; 4.3)	3.9 (3.3; 4.6)	<0.01	3.7 (3.2; 4.3)	3.9 (3.3; 4.7)	0.002	3.7 (3.2; 4.3)	3.9 (3.3; 4.6)	<0.01
**Covariates**									
Age (years)	10.2 (10.1; 10.3)	15.1 (15.0; 15.3)	<0.01	10.2 (10.1; 10.3)	15.1 (15; 15.3)	<0.01	10.2 (10.1; 10.3)	15.1 (15; 15.3)	<0.01
BMI (kg/m^2^)	16.7 (15.6; 18.4)	20.2 (18.7; 22.2)	<0.01	16.8 (15.6; 18.5)	20.4 (18.9; 22.3)	<0.01	16.7 (15.7; 18.4)	20 (18.6; 22.1)	<0.01
Fasting (yes)	237 (17.0)	649 (46.4)	<0.01 ^b^	121 (17.8)	309 (45.2)	<0.01 ^b^	116 (16.2)	341 (47.6)	<0.01 ^b^
Screen-time (high)	134 (9.6)	763 (55.3)	<0.01 ^b^	48 (7.0)	322 (47.8)	<0.01 ^b^	86 (12.0) ^§^	442 (62.5) ^§^	<0.01 ^b^
Energy intake (kcal)	1933 (1591; 2292)	2011 (1584; 2532)	<0.01	1798 (1486; 2124)	1734 (1360; 2115)	0.016	2061 (1705; 2447) ^§^	2361 (1884; 2866) ^§^	<0.01
Fat (%EI)	30.1 (26.7; 34.2)	30.5 (27.1; 34.8)	0.128	29.9 (26.1; 33.9)	30.0 (26.5; 34.2)	0.982	30.2 (27.3; 34.4)	30.9 (27.6; 35.3) ^§^	0.029
Carbohydrate (%EI)	54.1 (49.6; 58.0)	53.2 (48.6; 57.7)	0.004	54.3 (49.7; 58.4)	54.1 (49.1; 58.4) ^†^	0.696	53.7 (49.6; 57.5)	52.4 (47.7; 56.7)	<0.01
Protein (%EI)	14.5 (12.9; 16.0)	14.8 (13.1; 16.6)	<0.01	14.4 (12.8; 16.1)	14.5 (12.7; 16.3)	0.452	14.5 (13.1; 16.0)	15.1 (13.4; 16.8) ^§^	<0.01
*Study*									
GINI observation	452 (32.3)			221 (32.5)			231 (32.2)		
GINI intervention	437 (31.3)			224 (32.9)			213 (29.7)		
LISA	509 (36.4)			236 (34.7)			273 (38.1)		
*Region*									
Munich	807 (57.7)			389 (57.1)			418 (58.3)		
Leipzig	123 (8.8)			60 (8.8)			63 (8.8)		
Bad Honnef	65 (4.6)			29 (4.3)			36 (5.0)		
Wesel	403 (28.8)			203 (29.8)			200 (27.9)		
Parental education (High)	997 (71.3)			497 (73.0)			500 (69.7)		
Pubertal onset (Yes)	680 (48.6)			507 (74.4) ^†^			173 (24.1)		

Values are medians (25th percentile; 75th percentile) or counts (%); LDL = low-density lipoprotein; HDL = high-density lipoprotein; TAG = triglycerides; SFA = saturated fatty acids; TOTAL:HDL = total cholesterol to HDL ratio; MUFA = monounsaturated fatty acids; PUFA = polyunsaturated fatty acids; ^a^ tested by paired Wilcoxon signed rank test; ^b^ tested by McNemar’s chi-squared test; ^†^ value is significantly greater in females than in males at the respective time-point (*p*-value < 0.05, tested by Wilcoxon signed rank test or Fisher’s exact test); ^§^ value is significantly greater in males than in females at the respective time-point (*p*-value < 0.05, tested by Wilcoxon signed rank test or Fisher’s exact test).

**Table 2:** Single nutrient model: prospective associations of dietary fatty acid intakes at age 10 years (per IQR increase in %EI) with changes in blood lipid concentrations (mmol/L) from age 10 to 15 years.

Fatty Acids		ΔLDL			ΔHDL			ΔTAG			ΔTOTAL:HDL		
		β	95% CI	*p*-Value	β	95% CI	*p*-Value	β	95% CI	*p*-Value	β	95% CI	*p*-Value
**TOTAL**													
**SFA**	M_BASIC_	−0.038	−0.077; 0.001	0.057	0.005	−0.017; 0.027	0.653	**−0.038**	**−0.075; −0.001**	**0.042**	−0.030	−0.075; 0.015	0.196
	M_ADJ_	−0.036	−0.075; 0.003	0.068	0.005	−0.017; 0.027	0.638	**−0.038**	**−0.075; −0.001**	**0.042**	−0.029	−0.074; 0.016	0.209
**MUFA**	M_BASIC_	−0.012	−0.048; 0.024	0.519	0.011	−0.010; 0.031	0.306	−0.012	−0.046; 0.023	0.507	−0.017	−0.059; 0.025	0.428
	M_ADJ_	−0.011	−0.048; 0.025	0.534	0.011	−0.009; 0.031	0.297	−0.012	−0.046; 0.023	0.503	−0.017	−0.059; 0.025	0.430
***n*-3 PUFA**	M_BASIC_	−0.027	−0.058; 0.003	0.075	0.005	−0.012; 0.022	0.590	0.001	−0.028; 0.030	0.953	−0.017	−0.052; 0.018	0.342
	M_ADJ_	−0.027	−0.057; 0.003	0.082	0.005	−0.012; 0.022	0.540	0.000	−0.028; 0.029	0.981	−0.017	−0.053; 0.018	0.332
***n*-6 PUFA**	M_BASIC_	−0.003	−0.034; 0.028	0.866	0.015	−0.002; 0.032	0.089	0.009	−0.020; 0.039	0.525	−0.012	−0.048; 0.024	0.504
	M_ADJ_	−0.003	−0.034; 0.028	0.849	0.016	−0.002; 0.033	0.074	0.009	−0.021; 0.038	0.559	−0.013	−0.049; 0.023	0.465
**FEMALES**													
**SFA**	M_BASIC_	−0.048	−0.110; 0.014	0.131	0.012	−0.022; 0.045	0.491	−0.053	−0.100; −0.006	0.026	−0.057	−0.116; 0.002	0.060
	M_ADJ_	−0.047	−0.110; 0.015	0.139	0.012	−0.022; 0.045	0.484	−0.053	−0.100; −0.007	0.025	−0.057	−0.116; 0.002	0.060
**MUFA**	M_BASIC_	−0.004	−0.061; 0.053	0.888	0.020	−0.011; 0.050	0.211	−0.017	−0.060; 0.026	0.430	−0.026	−0.081; 0.028	0.342
	M_ADJ_	−0.005	−0.062; 0.053	0.876	0.020	−0.011; 0.050	0.209	−0.018	−0.061; 0.025	0.420	−0.027	−0.081; 0.028	0.336
***n*-3 PUFA**	M_BASIC_	−0.030	−0.078; 0.018	0.219	0.011	−0.015; 0.037	0.406	0.000	−0.036; 0.036	0.990	−0.032	−0.077; 0.014	0.173
	M_ADJ_	−0.030	−0.079; 0.018	0.214	0.011	−0.015; 0.037	0.403	0.000	−0.036; 0.036	0.998	−0.032	−0.078; 0.014	0.169
***n*-6 PUFA**	M_BASIC_	−0.020	−0.068; 0.028	0.409	0.019	−0.007; 0.044	0.148	0.016	−0.020; 0.052	0.390	−0.026	−0.071; 0.020	0.269
	M_ADJ_	−0.021	−0.069; 0.027	0.397	0.020	−0.006; 0.046	0.124	0.014	−0.022; 0.050	0.446	−0.028	−0.074; 0.018	0.231
**MALES**													
**SFA**	M_BASIC_	−0.029	−0.078; 0.019	0.237	−0.002	−0.031; 0.026	0.868	−0.016	−0.072; 0.04	0.577	0.002	−0.066; 0.070	0.962
	M_ADJ_	−0.026	−0.075; 0.022	0.282	−0.003	−0.031; 0.026	0.863	−0.015	−0.072; 0.041	0.591	0.003	−0.065; 0.071	0.929
**MUFA**	M_BASIC_	−0.021	−0.066; 0.025	0.372	0.000	−0.027; 0.027	0.975	−0.002	−0.055; 0.051	0.952	−0.001	−0.065; 0.063	0.972
	M_ADJ_	−0.019	−0.065; 0.026	0.400	0.001	−0.026; 0.027	0.964	−0.002	−0.056; 0.051	0.927	−0.001	−0.065; 0.063	0.968
***n*-3 PUFA**	M_BASIC_	−0.026	−0.064; 0.012	0.181	−0.003	−0.025; 0.02	0.818	0.005	−0.040; 0.049	0.826	0.001	−0.053; 0.055	0.971
	M_ADJ_	−0.025	−0.063; 0.013	0.199	−0.001	−0.023; 0.022	0.952	0.003	−0.042; 0.048	0.891	0.000	−0.054; 0.054	0.992
***n*-6 PUFA**	M_BASIC_	0.011	−0.029; 0.051	0.583	0.010	−0.013; 0.034	0.392	0.001	−0.045; 0.048	0.953	0.000	−0.056; 0.056	0.991
	M_ADJ_	0.013	−0.027; 0.052	0.534	0.012	−0.011; 0.036	0.299	−0.001	−0.047; 0.046	0.975	−0.001	−0.057; 0.055	0.971

IQR = interquartile range; %EI = % of total energy intake; M_BASIC_ = single nutrient model adjusted for study, region, sex (not in sex-stratified models), exact age at blood sampling, BMI at 10 years, total daily energy intake at 10 years, screen-time at 10 years, fasting status at blood sampling, and lipid concentration at 10 years; M_ADJ_ = single nutrient model further adjusted for pubertal onset and parental education; Δ = change from age 10 to 15 years; Significant associations marked in bold (*p*-value < 0.05 for total population analyses, *p*-value < 0.025 for sex-stratified analyses—Bonferroni correction for multiple testing: 0.05/2).

**Table 3:** Substitution model: prospective associations of fatty acids and carbohydrates (CHO) (when replacing SFA) at age 10 years (per IQR increase in %EI), with changes in blood lipid concentrations (mmol/L) from age 10 to 15 years.

Substituting Nutrient		ΔLDL			ΔHDL			ΔTAG			ΔTOTAL:HDL		
		β	95% CI	*p*-Value	β	95% CI	*p*-Value	β	95% CI	*p*-Value	β	95% CI	*p*-Value
**TOTAL**													
**MUFA**	M_BASIC_	−0.037	−0.085; 0.011	0.134	0.002	−0.025; 0.029	0.897	−0.041	−0.087; 0.005	0.077	−0.027	−0.083; 0.029	0.346
	M_ADJ_	−0.034	−0.082; 0.014	0.163	0.002	−0.025; 0.029	0.894	−0.041	−0.086; 0.005	0.081	−0.025	−0.082; 0.031	0.378
***n*** **-3 PUFA**	M_BASIC_	−0.027	−0.064; 0.011	0.164	−0.003	−0.024; 0.018	0.752	0.002	−0.033; 0.037	0.913	−0.008	−0.052; 0.036	0.715
	M_ADJ_	−0.026	−0.063; 0.012	0.179	−0.003	−0.024; 0.018	0.775	0.002	−0.034; 0.037	0.916	−0.008	−0.052; 0.036	0.723
***n*** **-6 PUFA**	M_BASIC_	0.018	−0.019; 0.056	0.341	0.016	−0.005; 0.037	0.143	0.019	−0.017; 0.055	0.303	−0.001	−0.045; 0.043	0.978
	M_ADJ_	0.017	−0.021; 0.054	0.384	0.016	−0.005; 0.038	0.129	0.018	−0.018; 0.054	0.324	−0.002	−0.047; 0.042	0.916
**CHO**	M_BASIC_	0.063	0.000; 0.127	0.050	−0.001	−0.036; 0.035	0.970	0.057	−0.003; 0.117	0.061	0.043	−0.031; 0.117	0.257
	M_ADJ_	0.060	−0.004; 0.123	0.064	−0.001	−0.037; 0.034	0.947	0.057	−0.003; 0.117	0.063	0.041	−0.033; 0.115	0.278
**FEMALES**													
**MUFA**	M_BASIC_	−0.053	−0.130; 0.024	0.175	0.005	−0.037; 0.046	0.825	−0.065	−0.122; −0.007	0.029	−0.054	−0.127; 0.020	0.153
	M_ADJ_	−0.052	−0.129; 0.025	0.188	0.005	−0.037; 0.046	0.824	−0.064	−0.122; −0.007	0.029	−0.053	−0.127; 0.020	0.154
***n*** **-3 PUFA**	M_BASIC_	−0.020	−0.080; 0.039	0.502	−0.004	−0.036; 0.028	0.822	0.002	−0.043; 0.047	0.928	−0.003	−0.060; 0.053	0.907
	M_ADJ_	−0.020	−0.079; 0.039	0.510	−0.004	−0.036; 0.028	0.788	0.003	−0.042; 0.048	0.895	−0.002	−0.059; 0.055	0.942
***n*** **-6 PUFA**	M_BASIC_	−0.004	−0.062; 0.054	0.896	0.015	−0.016; 0.046	0.341	0.034	−0.009; 0.078	0.125	−0.005	−0.060; 0.050	0.856
	M_ADJ_	−0.005	−0.063; 0.053	0.868	0.017	−0.014; 0.048	0.292	0.032	−0.012; 0.075	0.155	−0.008	−0.064; 0.047	0.774
**CHO**	M_BASIC_	**0.127**	**0.023; 0.231**	**0.017**	−0.003	−0.059; 0.053	0.916	**0.097**	**0.019; 0.175**	**0.015**	**0.114**	**0.014; 0.213**	**0.025**
	M_ADJ_	**0.125**	**0.021; 0.229**	**0.019**	−0.004	−0.060; 0.052	0.891	**0.098**	**0.020; 0.176**	**0.014**	**0.115**	**0.015; 0.215**	**0.024**
**MALES**													
**MUFA**	M_BASIC_	−0.027	−0.088; 0.033	0.373	−0.001	−0.036; 0.035	0.976	−0.015	−0.085; 0.055	0.668	−0.006	−0.091; 0.079	0.898
	M_ADJ_	−0.024	−0.084; 0.036	0.435	−0.001	−0.037; 0.035	0.948	−0.015	−0.085; 0.056	0.683	−0.004	−0.089; 0.082	0.934
***n*** **-3 PUFA**	M_BASIC_	−0.043	−0.091; 0.005	0.079	−0.008	−0.037; 0.020	0.570	0.006	−0.050; 0.062	0.833	−0.009	−0.076; 0.059	0.796
	M_ADJ_	−0.043	−0.090; 0.005	0.081	−0.007	−0.035; 0.021	0.630	0.005	−0.050; 0.061	0.851	−0.009	−0.077; 0.058	0.788
***n*** **-6 PUFA**	M_BASIC_	0.041	−0.009; 0.090	0.106	0.017	−0.012; 0.046	0.256	−0.004	−0.061; 0.054	0.898	0.001	−0.069; 0.071	0.985
	M_ADJ_	0.041	−0.008; 0.090	0.103	0.018	−0.011; 0.048	0.224	−0.004	−0.062; 0.053	0.880	0.000	−0.070; 0.070	0.995
**CHO**	M_BASIC_	0.017	−0.059; 0.093	0.658	−0.001	−0.046; 0.044	0.961	0.021	−0.068; 0.109	0.647	−0.008	−0.115; 0.099	0.884
	M_ADJ_	0.012	−0.064; 0.088	0.755	0.000	−0.045; 0.045	0.991	0.020	−0.069; 0.108	0.663	−0.011	−0.118; 0.096	0.842

IQR = interquartile range; %EI = % of total energy intake; M_BASIC_ = substitution model adjusted for study, region, sex (not in sex-stratified models), exact age at blood sampling, BMI at 10 years, total daily energy intake at 10 years, screen-time at 10 years, fasting status at blood sampling, lipid concentration at 10 years, and all energy-bearing nutrients except SFA; M_ADJ_ = substitution model further adjusted for pubertal onset and parental education; Δ = change from age 10 to 15 years; Significant associations marked in bold (*p*-value < 0.05 for total population analyses, *p*-value < 0.025 for sex-stratified analyses—Bonferroni correction for multiple testing: 0.05/2).

## Supplementary Materials

The following are available online at http://www.mdpi.com/2072-6643/9/2/127/s1. Table S1. Comparison of descriptive characteristics of participants included in analyses (data for exposure at age 10 years and outcome at age 10 years and age 15 years) and participants lost to follow-up (data only for exposure and outcome at age 10 years).
