# Supplementary material for: Dietary Fatty Acids and Changes in Blood Lipids during Adolescence: The Role of Substituting Nutrient Intakes
Source: Nutrients. 2017 Feb 11;9(2):127. doi: 10.3390/nu9020127 (PMC5331558; doi:10.3390/nu9020127)
Supplement: Supplementary file 1 [file nutrients-09-00127-s001.pdf]

Supplementary Materials: Dietary Fatty Acids and Changes in Blood Lipids during Adolescence: The Role of Substituting Nutrient Intakes

Carla Harris, Anette Buyken, Sibylle Koletzko, Andrea von Berg, Dietrich Berdel, Tamara Schikowski, Berthold Koletzko, Joachim Heinrich and Marie Standl

Table S1. Comparison of descriptive characteristics of participants included in analyses (data for exposure at age 10 years and outcome at age 10 years and age 15 years) and participants lost to follow-up (data only for exposure and outcome at age 10 years).

Variables	Included	Lost to follow-up	*p*-Value
N	1606	555	
BMI at 10 years [kg/m^2] [b]	16.8 (2.2)	17.1 (2.5)	0.008
Fasting at 10 years [yes] [a]	17.0	17.8	0.648
screen-time at 10 years [high] [a]	10.0	11.5	0.331
Energy intake at 10 years [kcal] [b]	1971.3 (548.2)	1932.3 (570.1)	0.160
Sex [female] [a]	48.7	50.8	0.403
Study [a]			
GINI observation	30.9	31.7	
GINI intervention	34.2	29.7	
LISA	34.9	38.6	0.122
Region [a]			
Munich	57.0	57.7	
Leipzig	8.9	7.2	
Bad Honnef	4.7	5.8	
Wesel	29.4	29.4	0.509
Parental education level [high] [a]	70.9	66.6	0.065
Pubertal onset 10 years [yes] [a]	71.6	67.6	0.092

[a] Presented as percentage and tested by Fisher's exact test (binary variables) or Pearson's Chi-squared test (variables with more than 2 levels); [b] Presented as mean (standard deviation) and tested by *t*-test.
